# A Novel Synthetic Smoothened Antagonist Transiently Inhibits Pancreatic Adenocarcinoma Xenografts in a Mouse Model

**DOI:** 10.1371/journal.pone.0019904

**Published:** 2011-06-15

**Authors:** Martin F. Strand, Steven R. Wilson, Jennifer L. Dembinski, Daniel D. Holsworth, Alexander Khvat, Ilya Okun, Dirk Petersen, Stefan Krauss

**Affiliations:** 1 Unit for Cell Signalling, Institute for Microbiology, Oslo University Hospital, Rikshospitalet, Oslo, Norway; 2 ChemDiv Inc., San Diego, California, United States of America; 3 Department of Chemistry, University of Oslo, Oslo, Norway; National Cancer Institute, United States of America

## Abstract

**Background:**

Hedgehog (Hh) signaling is over-activated in several solid tumors where it plays a central role in cell growth, stroma recruitment and tumor progression. In the Hh signaling pathway, the Smoothened (SMO) receptor comprises a primary drug target with experimental small molecule SMO antagonists currently being evaluated in clinical trials.

**Principal Findings:**

Using Shh-Light II (Shh-L2) and alkaline phosphatase (AP) based screening formats on a “focused diversity” library we identified a novel small molecule inhibitor of the Hh pathway, MS-0022 (2-bromo-*N*-(4-(8-methylimidazo[1,2-*a*]pyridin-2-yl)phenyl)benzamide). MS-0022 showed effective Hh signaling pathway inhibition at the level of SMO in the low nM range, and Hh pathway inhibition downstream of Suppressor of fused (SUFU) in the low µM range. MS-0022 reduced growth in the tumor cell lines PANC-1, SUIT-2, PC-3 and FEMX *in vitro.* MS-0022 treatment led to a transient delay of tumor growth that correlated with a reduction of stromal Gli1 levels in SUIT-2 xenografts *in vivo*.

**Significance:**

We document the *in vitro* and *in vivo* efficacy and bioavailability of a novel small molecule SMO antagonist, MS-0022. Although MS-0022 primarily interferes with Hh signaling at the level of SMO, it also has a downstream inhibitory effect and leads to a stronger reduction of growth in several tumor cell lines when compared to related SMO antagonists.

## Introduction

The Hedgehog (Hh) signaling pathway is one of the key regulators in vertebrate development and is highly conserved among species from fruit flies to humans [1]–[4]. It is also one of the key pathways that regulate stem cells in the adult body [5]. Aberrant Hh signaling has been associated with a number of human tumors where the pathway has been implicated in tumor growth, malignancy, metastasis, and cancer stem cells [6]–[9]. Thus, the Hh pathway has become a focus for drug discovery and development [10]–[15].

The Hh pathway is unusual by several means, and central aspects of its functioning remain to be explored. The morphogens IHH, DHH and SHH interact with the 12-pass transmembrane receptor Patched (PTCH). PTCH inhibits the physically separate 7-pass transmembrane receptor Smoothened (SMO) by gating the movement of SMO into cilia. Evidence suggests, that upon Hh binding, PTCH leaves the shaft of the primary cilium which allows SMO to enter from its inactive endosomal state into cilia [16]–[18]. Furthermore, it has been proposed that SMO exists in an inactive and active state [19], [20] that may be regulated through a hypothesized sterol-like small molecule [4], [19], [21]. SMO migration into the primary cilium is followed by the inactivation of Suppressor of fused (SUFU) [22]. Current data suggest that SUFU, being a part of a multiprotein complex that also includes β-arrestin, KIF3a and IFT88, impedes the nuclear localization of GLI proteins [16], [17], [22]. In addition it may act as a nuclear co-repressor [23]. SUFU is ubiquitinated upon the activation of Hh signaling which initiates its degradation in the proteasomes [24] leading to the release of GLI2/3 into the nucleus where they regulate transcription of downstream target genes including the activating transcription factor GLI1. Although GLI1 presence in the nucleus is primarily a consequence of active Hh signaling, it can be attenuated by other signaling pathways [25].

There are several key mechanisms in tumorigenesis that may involve Hh/GLI signaling [11], [13]; first, inactivating mutations in the negative regulators PTCH or SUFU, or activating mutations in the positive regulator SMO cause pathway activation in a cell-autonomous and Hh ligand independent manner [5], [26]–[28]; secondly, ligand-dependent autocrine mechanisms in which cancer cells both secrete and respond to Hh ligands causing cell-autonomous pathway activation [29], [30]; thirdly, paracrine mechanisms in which stromal cells are induced by Hh producing cancer cells [31]–[34]. Both autocrine and paracrine effects can lead to heterogeneity with respect to Hh pathway activity within a tumor [35]. Several SMO antagonists have been developed and early data show clinical efficacy in selected tumors [36]. However, there has been some debate whether the *in vivo* growth inhibition observed for Hh antagonists is due to inhibition of autocrine or paracrine Hh signaling. Several recent studies suggest that the primary role of Hh inhibition in Hh secreting tumors may be due to the inhibition of paracrine signaling involving tumor-stroma interactions [33], [37]–[41]. In particular, tumor derived SHH has been shown to promote desmoplasia in pancreatic cancer [42], where the induced stroma in combination with poor vascularization may act as a barrier that is linked to a poor response to chemotherapy [40], [41].

Following the identification of cyclopamine as a natural SMO inhibitor [43]–[45], several Hh pathway antagonists have been reported that either act at the level of SMO [46], GLI1 [47], or other parts of the pathway [10], [13], [36]. Among these inhibitors, some have been progressed to clinical trials. One of these, GDC-0449 [15], [34], [48], is currently in several phase I and phase II clinical trials for various types of cancers, including pancreatic cancer (trial ID: NCT01064622 and NCT00878163). Also, the cyclopamine derivative IPI-926 [14] has been through a phase I clinical trial in patients with non-disclosed advanced and/or metastatic solid tumors, and is currently in a phase Ib/II clinical trial in patients with untreated metastatic pancreatic cancer (trial ID: NCT01130142).

Here, we describe the identification and evaluation of a novel small molecule SMO antagonist, MS-0022. MS-0022 displays a differential efficacy on various solid tumors *in vitro* and on PANC-1 and SUIT-2 xenografts *in vivo*. The reported findings are a further confirmation of the potential of small molecule Hh antagonists as anticancer agents.

## Results

### Identification of the novel Hh antagonist MS-0022

**Figure 1:**
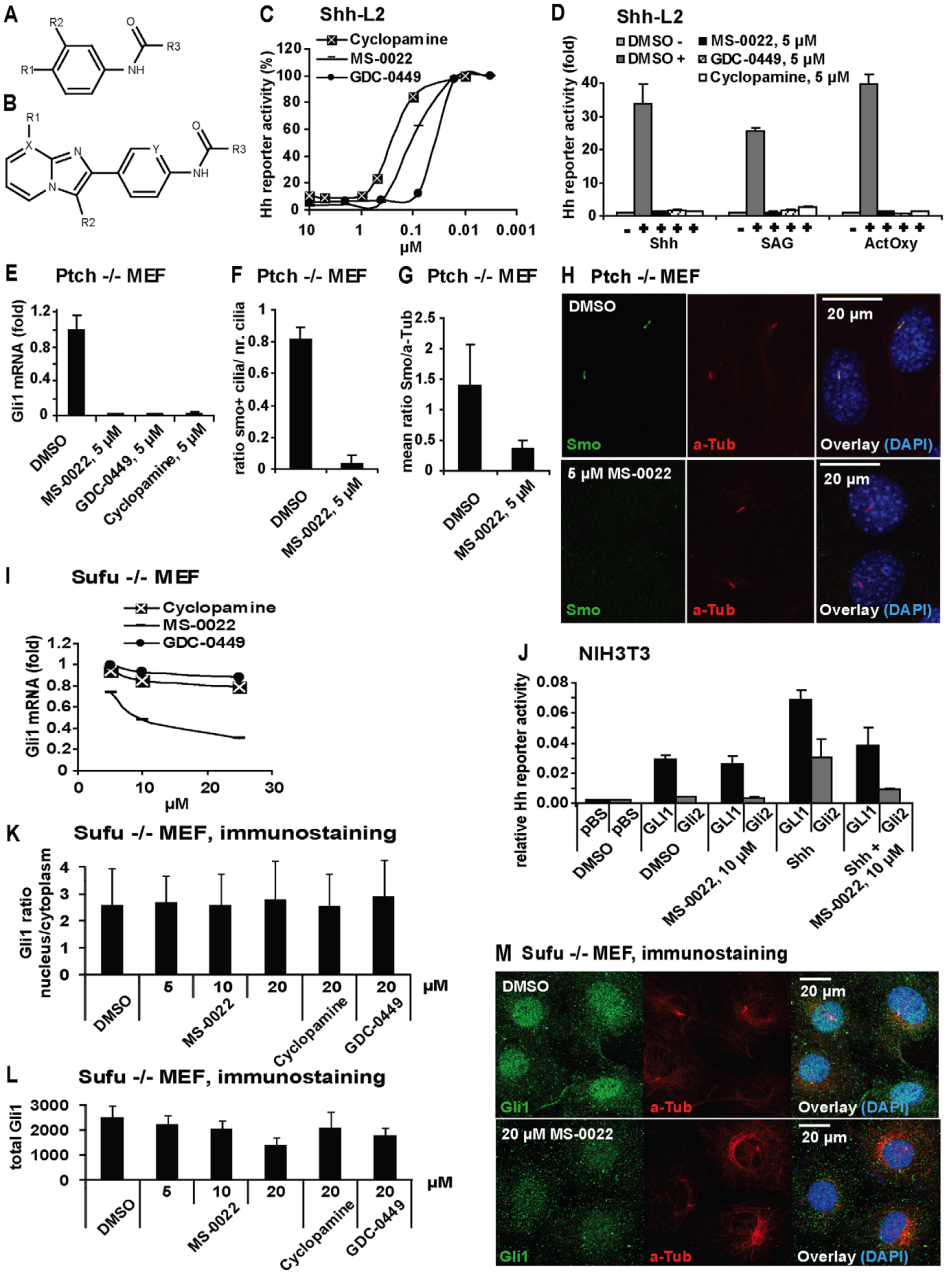
Identification and activity of MS-0022. A) Structure for Table 1. B) Structure for Table 2. C) Dose response curves of indicated compounds in Shh activated Shh-L2 cells after 48 hours treatment (n = 8). D) Shh-L2 cells activated with Shh, 200 nM SAG and ActOxs (5 µM+5 µM) treated with indicated compounds at 5 µM for 48 hours. Graph displays fold Hh reporter activity with standard deviation (SD) (n = 3). The basal activity (DMSO) was set as  = 1. E) Normalized (GADPH) Gli1 mRNA expression in Ptch−/− MEF after 48 hours of treatment with indicated compounds at 5 µM. Graph displays fold expression compared to DMSO control, with SD (n = 3). F–H) Immunostaining of Ptch −/− MEF cells treated with MS-0022 or DMSO control for 24 hours (anti-smoothened (Smo) in green, anti acetylated Tubulin (aTub) in red, DAPI in blue). Treated and control culture slides (n = 3) were stained and 3 regions on each slide was imaged and analyzed. F) Graph displaying the ratio of Smo positive cilia versus total number of cilia in stained cells by eye count, with SD. G) Graph displaying the ratio of mean ciliary intensity of Smo staining versus aTub as quantified using ImageJ software, with SD. I) Dose response curve of MS-0022, GDC-0449 and cyclopamine by normalized (GAPDH) Gli1 mRNA expression in Sufu −/− MEF after 72 hours treatment (n = 3). J) NIH3T3 cells cotransfected with GliBS-Luc in combination with pBluescript (pBS), FL-GLI1-HA or Gli2-GFP, were treated with DMSO, 50% Shh conditioned medium, 10 µM MS-0022 and a combination of Shh and 10 µM MS-0022 for 48 hours. Graph displays relative Hh pathway activity (reporter firefly luciferase normalized for renilla transfection control), with SD (n = 3). K–M) Immunostaining of Sufu−/− MEF cells treated with DMSO control, MS-0022 (5, 10 and 20 µM), cyclopamine (20 µM) and GDC-0449 (20 µM) for 48 hours (n = 3). K) Graph displaying ratio of nuclear Gli1 staining intensity versus cytoplasmic Gli1 staining intensity, with SD. L) Graph displaying sum of nuclear and cytoplasmic Gli1 staining intensity in treated cells, with SD.

To identify novel antagonists to Hh signaling, a focused diversity library of 12,000 compounds (10 µM) was screened using C3H10T1/2 cells induced by recombinant human SHH and employing an alkaline phosphatase (AP) readout in high throughput format [49], followed by a verification step using Shh-L2 cells. MS-0022 (2-bromo-*N*-(4-(8-methylimidazo[1,2-*a*]pyridin-2-yl)phenyl)benzamide), was identified as a potent Hh pathway antagonist with an IC_50_ of 100 nM in Shh-L2 cells. The structure of MS-0022 was confirmed by ^1^H and ^13^C NMR (Table S1 and Figure S1). In order to explore the parts of the core structure required for activity in MS-0022, a small scale broad structural analysis was performed based on activity inhibition in Shh-L2 cells. As shown in Table 1, a deletion of the 2-bromophenyl or the Imidazo[1,2-a]pyridine moiety of MS-0022 led to a substantial loss of activity (for the structure backbone see Figure 1A). The activity was partially retained when replacing the Imidazo[1,2-a]pyridine system with a napthlene-2-ylcarbamoyl system (MS-0018) or a 6-morpholinopyridazin-3-yl system (MS-0015). A further focused structural analysis of a limited number of MS-0022 analogs is shown in Table 2 (for the base structure see Figure 1B).

**Table 1:** Activity of MS-0022 and deletion/substitution analogs (See structure in figure 1A).

ID	R1	R2	R3	MW	IC50 (Shh-L2, nM)
MS-0022	8-methylimidazo[1,2-a]pyridine	–	2-bromophenyl	406.3	100
MS-0011	8-methylimidazo[1,2-a]pyridine	–	Methyl	265.3	>20,000
MS-0012	8-methylimidazo[1,2-a]pyridine	–	2-propyl	293.4	>20,000
MS-0013	–	*O*-Amide	2-bromophenyl	319.2	>20,000
MS-0014	Propyl	–	2-bromophenyl	318.2	10,000
MS-0015	6-morpholinopyridazin-3-yl	–	2-bromophenyl	439.3	800
MS-0016	(6-phenylpyrimidin-4-yl)amino	–	2-bromophenyl	445.3	2000
MS-0017	naphthalen-2-ylcarbamoyl	–	2-bromophenyl	445.3	280
MS-0018	2-benzylidenehydrazinecarbonyl	–	2-bromophenyl	442.3	5000

Table depicting molecular weight and bological activity (nM IC50) of deletion or substitution analogs of MS-0022 as measured by pathway inhibition in Shh induced Shh-L2 cells.

**Table 2:** Activity analysis of MS-0022 chemotype (See structure in figure 1B).

ID	R1	R2	R3	X	Y	MW	IC50 (Shh-L2,nM)	IC50 (nM, BODIPY- cyclopamine inhib.)	cLogP	PSA
MS-0022	CH3	H	2-bromophenyl	C	C	406.3	100	259	5.44	46.4
MS-0030	CH3	CH3	2-bromophenyl	C	C	420.3	630	–	5.86	46.4
MS-0031	CH3	H	2-bromophenyl	C	N	407.3	2300	–	3.88	59.29
MS-0032	CH3	H	2-fluorophenyl	C	C	345.4	161	93	4.39	46.2
MS-0033	H	H	4-methoxyphenyl	C	C	343.4	181	287	4.24	55.63
MS-0034	–	H	2-methoxyphenyl	N	C	344.4	>10,000	–	3.21	68.5
MS-0035	–	H	4-methoxyphenyl	N	C	344.4	877	–	3.1	68.52
MS-0036	–	H	3,5-dimethylphenyl	N	C	342.4	3,700	–	4.06	59.29
MS-0037	–	H	3-chlorophenyl	N	C	348.8	1,870	–	3.87	59.29
MS-0038	–	H	2-trifluoromethylphenyl	N	C	382.3	1,150	–	2.98	59.3

Biological activity of side group analogues of MS-0022 measured by pathway inhibition in Shh induced Shh-L2 cells and by BODIPY-cyclopamine competition, including cLogP (computed LogP, octanol/water partition coefficient) and polar surface area (PSA).

Changing R2 from a hydrogen to a methyl group reduced activity 6-fold (MS-0030). Incorporation of a nitrogen atom in position Y reduced activity 23-fold (MS-0031). Substitution of R3 with a 2-fluorophenyl group reduced activity 1.6-fold (MS-0032). Interestingly, if R1 was replaced with a hydrogen and R3 with 4-methoxyphenyl, the activity dropped 1.8-fold (MS-0033). If R1 of MS-0033 was replaced with a nitrogen atom in the ring (MS-0035), the activity dropped an additional 8-fold. In general, a nitrogen atom in the 8-position of the imidazo[1,2-a]pyridine ring system caused a significant reduction in activity. Furthermore, a nitrogen atom placed in position Y impacted the activity negatively.

A dose response curve of MS-0022 in Shh activated Shh-L2 cells, is shown in Figure 1C, using cyclopamine and GDC-0449 as a comparison. MS-0022 exhibited an IC_50_ of 100 nM, while cyclopamine exhibited an IC_50_ of 210 nM and GDC-0449 an IC_50_ of 30 nM.

To ascertain that the compounds interacted at the level of SMO, the most potent compounds, MS-0022, MS-0032 and MS-0033 were shown to compete with BODIPY-cyclopamine with IC_50_^'^s of 259, 93 and 287 nM respectively (Table 2). Thus, MS-0022 and analogs of MS-0022, as well as GDC-0449, inhibit Hh signaling at the level of SMO. MS-0022 inhibited SMO with similar affinity as cyclopamine.

### MS-0022 blocks Hh signaling at the level of SMO, but has additional activity downstream of SUFU

MS-0022 was shown to compete with BODIPY-cyclopamine, and the observed effect on C3H10T1/2 cells and Shh-L2 cells suggested that MS-0022 blocks Hh signaling at the level of SMO. However, to further verify the function of MS-0022 as a SMO blocker, the activity of MS-0022 was evaluated in experiments that explore the various modes of Hh signaling induction in mouse cell lines. Upstream of Gli, Hh signaling can be induced by the Hh morphogens; by functional loss of Ptch, as present in the constitutively active Ptch−/− mouse embryonic fibroblast (MEF) cell line; by a combination of the activating oxysterols hydroxycholesterol 20-alpha and hydroxycholesterol 22S (ActOXS) and by the SMO agonist SAG and by Sufu inactivation, as present in Sufu−/− MEFs. As seen in Figure 1D, MS-0022 blocked Hh pathway induction in Shh, SAG and ActOXS induced Shh-L2 cells similarly to GDC-0449 and cyclopamine. MS-0022 also reduced Gli1 mRNA expression in Ptch−/− MEFs, similar to GDC-0449 and cyclopamine (Figure 1E). Cyclopamine blocks Smo function within primary cilia without blocking its translocation to the cilia. In contrast, MS-0022 effectively blocked ciliary accumulation of Smo (Figure 1F-H), indicating an effect on the level of Smo similar to GDC-0449, but divergent from cyclopamine. Primary cilia in reporter cells were identified using an antibody against the ciliary maker acetylated tubulin. Acetylation of tubulin is found in stabilized microtubular structures like cilia and mitotic spindles, and has effectively been used as a marker for cilia in various studies [50], [51].

As expected for a SMO antagonist, cyclopamine and GDC-0449 exhibited little inhibitory effect on Hh signaling downstream of Smo in Sufu−/− MEFs. In contrast, MS-0022 reduced relative Gli1 mRNA levels Sufu−/− MEFs by 50% at a dose of 10 µM (Figure 1I). Further downstream, at the level of Gli1 or Gli2, Hh pathway inhibitory effect by MS-0022 was not detected as indicated by the lack of inhibition of a forced expression of either of the two transcription factors with 10 µM of MS-0022 (Figure 1J). MS-0022 was able to show partial inhibition (Figure 1J) of Gli1 or Gli2, only when the Hh signaling pathway was further activated by Shh. In order to further elucidate the activity of MS-0022, Sufu−/− MEF cells that have been treated with MS-0022, cyclopamine or GDC-0449 were stained with an antibody recognizing Gli1. The intensity of Gli1 staining in the nucleus and the cytoplasm was measured, and neither of the treatments resulted in a shift in the ratio of Gli1 in the nucleus versus the cytoplasm (Figure 1K). Thus the reduction of Gli1 mRNA levels in the Sufu−/− MEF cells by MS-0022 was not a consequence of Gli1 relocation in the cell. However, while the treatment with 20 µM MS-0022 did not alter the ratio of nuclear versus cytoplasmic Gli1, the total level of Gli1 in the cells was reduced (Figure 1 L–M), correlating well with the reduction in Gli1 mRNA. At the same dose GDC-0449 also reduced Gli1 levels in the cells, but to a lesser extent than MS-0022 (Figure 1L), while cyclopamine had no significant effect.

We conclude that MS-0022 acts at the level of SMO blocking its ciliary transport in the nanomolar range. An additional inhibitory effect of MS-0022 on Hh signaling downstream of Sufu that required a dose in the 10–20 micro molar range is linked to a reduction of Gli1 protein levels.

### MS-0022 blocks tumor growth in pancreatic adenocarcinoma, prostate carcinoma and melanoma cell lines *in vitro*

To test *in vitro* efficacy of MS-0022, we profiled the presence of central components of the Hh signaling pathway in the pancreas adenocarcinoma cell lines PANC-1 and SUIT-2, the prostate cancer cell line PC-3, and the melanoma cell line FEMX by real time PCR (Table 3). Although all cell lines expressed detectable levels of GLI1 mRNA, the level of expression varied, as did other components of the Hh signaling pathway. However, the clear presence of the direct Hh downstream marker PTCH1 in all cell lines, indicated Hh/GLI1 pathway activity.

**Table 3:** Expression profile, Tumor cell lines.

	PTCH1	SMO	GLI1	GLI2	GLI3	SHH	HIP	SUFU
PANC-1	+++	+	+++	+++	+	+++	+/−	+++
SUIT-2	++	++	++	++	**nt**	+++	**nt**	**nt**
PC-3	++	++	++	+	+	+++	+	+
FEMX	++	+	+	+	−	−	−	+

Expression analysis of tumor cell lines according to the PCR cycle where amplification was detected, starting with 1 µg mRNA (+++ = 20–25, ++ = 25–30, + = 30–35, − = >35 and nt  =  not tested).

**Figure 2:**
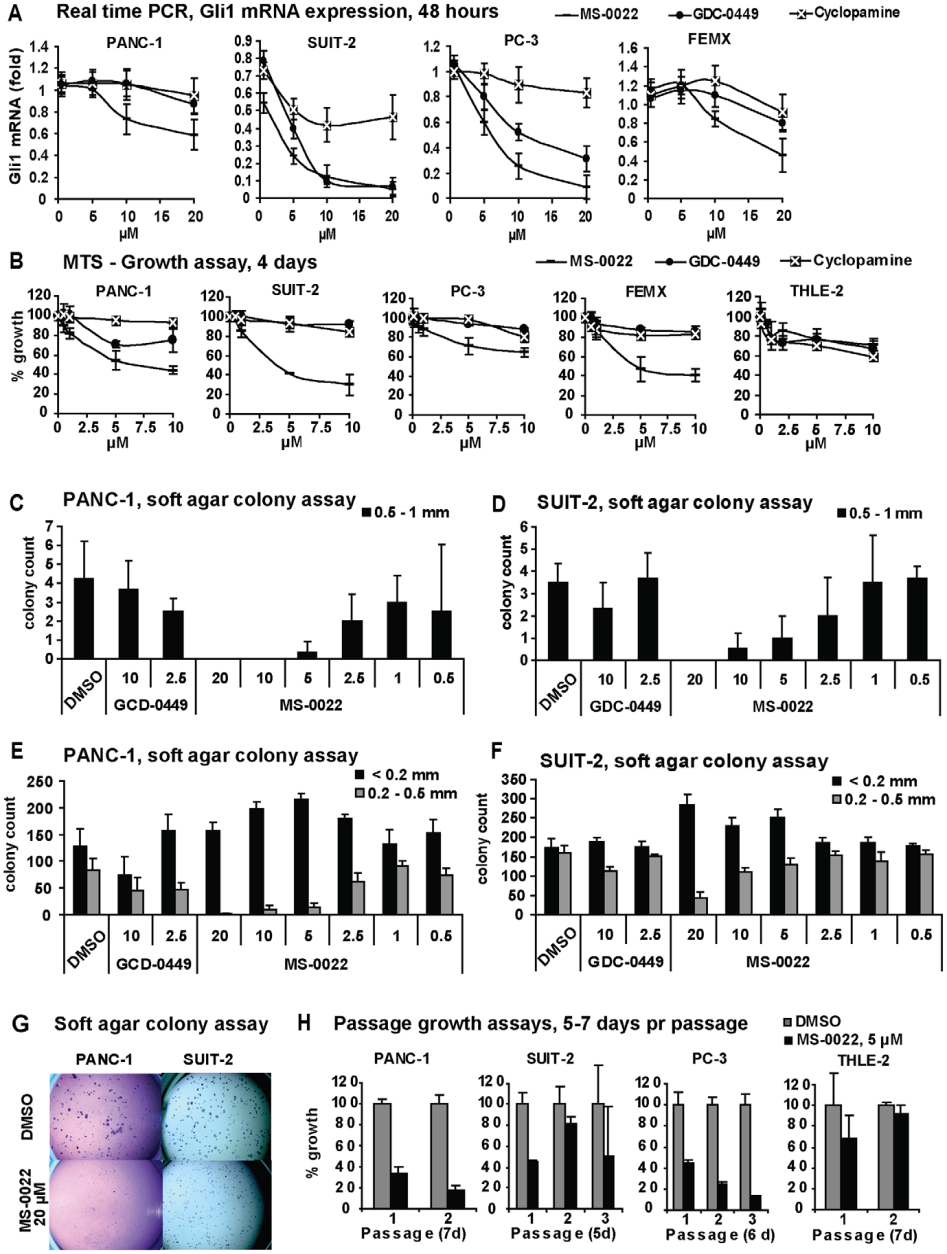
*In vitro* activity in tumor cell lines and a control cell line. A) Graphs displaying dose response curves of normalized (GAPDH) Gli1 mRNA expression in indicated tumor cell lines after 48 hour treatment of MS-0022, cyclopamine and GDC-0449. Graphs include standard deviation (n = 3). B) Graphs displaying 4 day dose response curves of cell growth as a % growth of DMSO control (MTS) for PANC-1, SUIT-2, PC-3, FEMX and THLE-2 cells treated with MS-0022, GDC-0449 and cyclopamine at the indicated dose, with SD (n = 8) C-G) Soft agar colony formation assay of PANC-1 (21 days) and SUIT-2 (14 days) cells treated with DMSO or MS-0022 and GDC-0449 at the indicated doses (n = 3). Each well was imaged and colonies were counted by eye according to size. Graphs displaying the colony count of larger colonies (0.5–1 mm) are shown in C) for PANC-1 and D) for SUIT-2, with SD. Graphs displaying the colony count of medium (0.2–0.5 mm) and small (<0.2 mm) colonies are shown in E) for PANC-1 and F) for SUIT-2, with SD. H) Passage growth assays with PANC-1, SUIT-2, PC-3 and THLE-2 cell lines treated with DMSO control or 5 µM MS-0022 for 2–3 passages of 5–7 days, displayed as % growth of DMSO control, with SD (n = 3).

To determine if GLI1 mRNA levels in the tumor cells could be reduced by MS-0022, real time PCR was carried out on cells treated with different doses of MS-0022, cyclopamine and GDC-0449 for 48 hrs (Figure 2A). In parallel, growth inhibition was measured by MTS (Figure 2B). For the PANC-1 cell line, growth inhibition and reduction of GLI1 mRNA levels upon treatment, correlated well at 10 µM for all three compounds. At 5 µM, however, MS-0022 and GDC-0449 reduced growth without reducing GLI1 mRNA levels. In the SUIT-2 cell line, MS-0022, cyclopamine and GDC-0449 all reduced GLI1 mRNA levels, but only MS-0022 reduced growth. For the PC-3 cell line, both MS-0022 and GDC-0449 reduced GLI1 mRNA levels, although growth was only reduced by MS-0022. For the FEMX cell line, the growth and GLI1 mRNA levels correlated well at 10 µM, but not at 5 µM. In conclusion, using the SMO antagonists GDC-0449 and cyclopamine, no correlation between growth inhibition and reduction of GLI1 mRNA levels could be detected in the four tumor cell lines PANC-1, SUIT-2, PC-3 and FEMX. However, a correlation between growth inhibition and GLI1 mRNA levels were apparent at a dose of 10 µM MS-0022 across all four tumor cell lines. The data set is consistent with an additional Hh pathway inhibitory effect of MS-0022 downstream of SMO/SUFU that requires relative higher doses of the compound as compared to direct SMO inhibition. As seen in Figure 2B, both CDG-0449 and cyclopamine did not lead to more than 30% growth reduction in the tested cell lines PANC-1, SUIT-2, PC-3 and FEMX during a 4 day exposure to 10 µM compound in a MTS assay. In contrast, at the same dose, MS-0022 reduced growth from 40%–70% in the same cell lines (Figure 2B). An immortalized, non-tumorigenic hepatocyte cell line, THLE-2, was included as a control, and the THLE-2 cells responded with a 25%–30% growth reduction to 10 µM compound exposure possibly indicating a weak Hh dependency in this control cell line (Figure 2B).

To address growth inhibition in a model relevant to xenograft studies, PANC-1 and SUIT-2 cells were seeded in a soft agar colony forming assay. A dose response curve was generated for MS-0022, while using GDC-0229 and cyclopamine as controls. For both cell lines, treatment with MS-0022 led to a reduction in large (Figure 2C–D, and G) and medium sized colonies in a dose dependant manner (Figure 2E–F, and G). Also upon MS-0022 treatment, an increase in the number of small colonies was observed, indicating that the reduced growth of the small and medium sized colonies is linked to decreased proliferation rather than apoptosis. The cyclopamine control was excluded from the dataset due to problems with crystallization of cyclopamine in the soft agar.

Long term growth assays with 2–3 serial passages (5–7 days per passage) further confirmed the efficacy of MS-0022 on the tested PANC-1, SUIT-2, PC-3 and control THLE-2 cell lines (Figure 2H). In the presence of 5 µM MS-0022, there was an initial growth reduction in the first passage of the control THLE-2 cells, but by the second passage the growth was not affected by the treatment. In contrast, the growth was reduced by 80% in PANC-1 cells and PC-3 cells after passage 2. Serial passage growth reduction was not significant for passage 2 and 3 in the SUIT-2 cells.

### Pathway specificity

**Figure 3:**
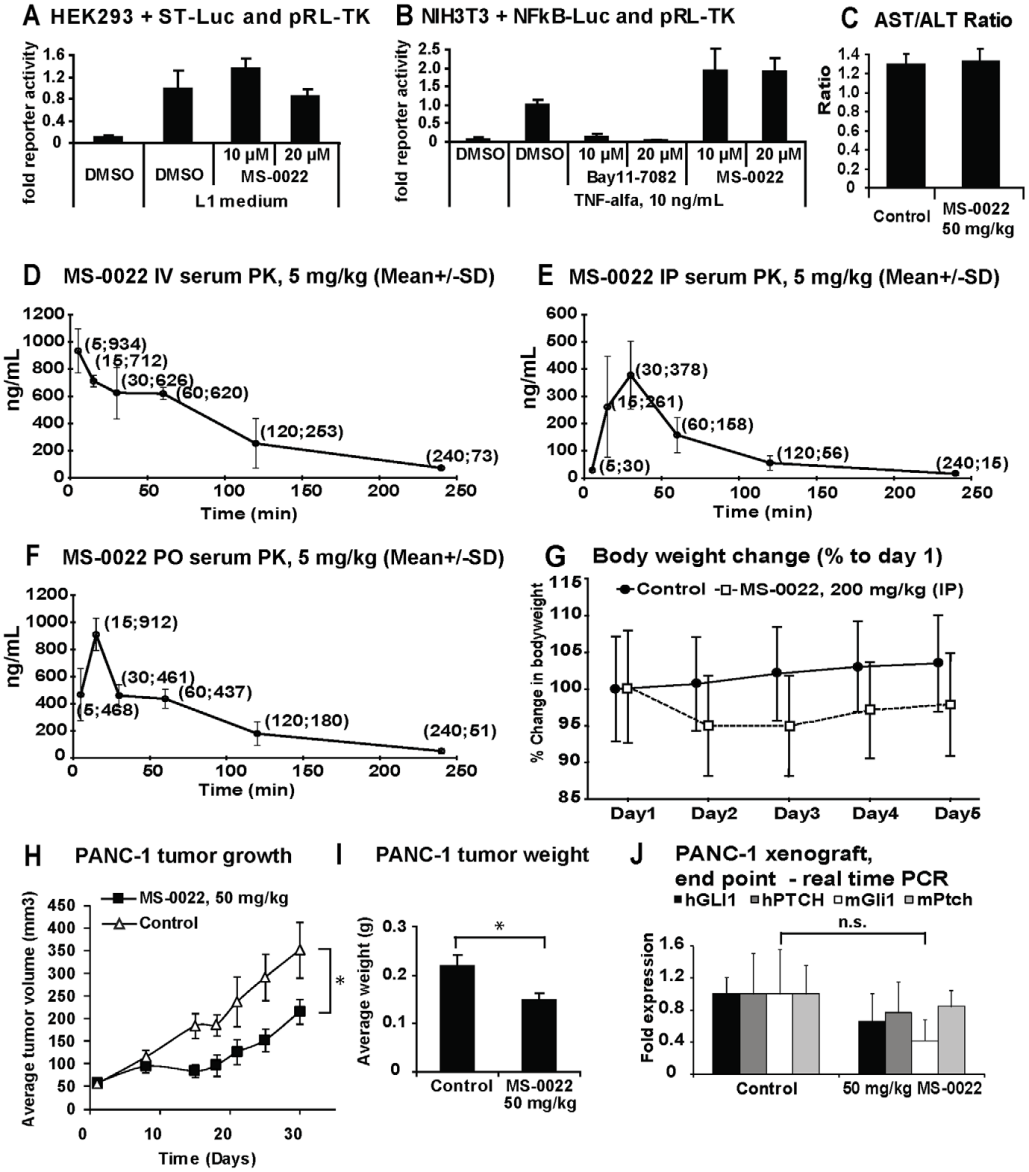
*In vitro* MS-0022 pathway selectivity, *in vivo* tolerance and distribution, and activity in a PANC-1 xenograft model. A) HEK293 cells cotransfected with SuperTop-luc (ST-Luc) and pRL-TK, were treated with control medium and L1 medium +/− 10 and 20 µM MS-0022 for 24 hours. Graph displays fold reporter activity with standard deviation (n = 3). B) NIH3T3 cells cotransfected with NFkB-luc and pRL-TK, treated with control medium and 10 ng/mL TNF-α +/− 10 and 20 µM NFkB inhibitor Bay11-7082 or 10 and 20 µM MS-0022 for 24 hours. Graph displays fold reporter activity with SD (n = 3). C) Graph depicting average AST/ALT ratio from mouse blood at end of animal treatment in the PANC-1 xenograft. D–F) Blood distribution curves after IV, IP and PO administration of 5 mg/kg MS-0022 in mice. Graphs display mean dose (ng/mL by time) +/− SD. G) Body weight change in animals treated with 200 mg/kg MS-0022 over 5 days, with SD (n = 5). H–J) Analysis of subcutaneous PANC-1 tumors in mice treated with solvent control or 50 mg/kg MS-0022 for 21 days (n = 6) by IP administration. H) Growth curves of PANC-1 tumors in mice after 30 days of treatment, showing average tumor volume (mm^3^) with standard error of the mean (SEM). I) Graph displaying average PANC-1 tumor weight at end of treatment, with SD. J) Graph displays normalized (GUSB) fold expression of mouse and human Gli1 and Ptch in treated tumors, with SD (n = 3). *  = P value <0.05. (n.s.  = P value >0.05).

To address possible effects of MS-0022 on other central signaling pathways, we analyzed whether MS-0022 affected Wnt and TNF-α signaling using firefly luciferase reporter assays. As seen in Figure 3A, MS-0022 did not significantly block L1 medium induced Wnt signaling in HEK293 cells, nor did it block TNF-α induced NfkB signaling in NIH3T3 cells at 10 µM and 20 µM (Figure 3B). Instead, MS-0022 led to a slight increase of NFkB signaling (Figure 3B). In addition, MS-0022 was tested at 10 µM in a Millipore diversity panel comprised of 58 kinases. MS-0022 did not significantly inhibit the activity of any of the tested kinases (Table 4).

**Table 4:** Kinase activity in presence of 10 µM MS-0022 in a kinaseprofiler diversity array.

kinase:	Activity %	kinase:	Activity %	kinase:	Activity %
Abl(h)	96	EphB4(h)	99	NEK2(h)	127
ALK(h)	95	Fyn(h)	100	p70S6K(h)	94
AMPK(r)	98	GRK5(h)	119	PAK2(h)	93
ASK1(h)	91	GSK3β(h)	88	PDGFRβ(h)	108
CaMKI(h)	106	IGF-1R(h)	105	Pim-1(h)	103
CDK1/cyclinB(h)	110	IKKα(h)	103	PKA(h)	100
CDK2/cyclinA(h)	98	IRAK4(h)	95	PKBα(h)	107
CDK7/cyclinH/MAT1(h)	103	JAK2(h)	110	PKCα(h)	106
CDK9/cyclin T1(h)	98	JNK3(h)	71	PKCθ(h)	103
CHK1(h)	84	KDR(h)	103	PKG1α(h)	94
CK1γ1(h)	93	LOK(h)	93	Plk3(h)	107
CK2α2(h)	109	Lyn(h)	103	PRAK(h)	71
cKit(h)	109	MAPK2(h)	101	ROCK-I(h)	103
c-RAF(h)	106	MAPKAP-K2(h)	92	Rse(h)	107
cSRC(h)	83	MEK1(h)	73	Rsk1(h)	91
DRAK1(h)	97	MKK7β(h)	109	SAPK2a(h)	102
DYRK2(h)	97	MLK1(h)	103	SRPK1(h)	102
eEF-2K(h)	99	Mnk2(h)	90	TAK1(h)	98
EGFR(h)	84	MSK2(h)	92		
EphA5(h)	112	MST1(h)	94		

Millipore diversity panel - percent activity of tested kinases in the presence of 10 µM MS-0022.

### Efficacy and bioavailability of MS-0022 in an *in vivo* pancreatic adenocarcinoma xenograft model

In preparation for *in vivo* xenografts, the bioavailability of MS-0022 in mouse plasma was evaluated. The maximum concentration (Cmax) of the compound in plasma was 934 ng/mL upon a 5 mg/kg IV injection. For a 5 mg/kg IP injection, it was 378 ng/mL and after 5 mg/kg PO administration, it was 912 ng/mL (Figure 3D–F and Table 5). Per oral bioavailability was calculated to be 98%. The half-life (T_1/2_) after MS-0022 administration was between 55 and 60 minutes, indicating that MS-0022 has a moderate stability in plasma. Compound concentration in mouse liver tissue 2 hours after IP administration was 244 ng/g. Thus, MS-0022 readily enters tissue from the plasma. Overall, the compound exhibited good exposure and moderate stability in plasma.

**Table 5:** Pharmacokinetics of MS-0022.

	Cmax, ng/ml	AUClast ng*min/ml	AUCinf ng*min/ml	T1/2, min	Kel, min-1
IV	934.3	87683.5	93897.6	59.27	0.0117
IP	377.7	25025.0	26237.8	54.83	0.0126
PO	912.0	64177.5	68526.0	59.10	0.0117

Pharmacokinetic profile of MS-0022 by IV, IP and PO administration in mice. The table displays the maximum concentration (Cmax), total drug exposure (AUClast) and (AUCinf), half-life (T1/2) and terminal slope (Kel).

Next the *in vivo* tolerability of MS-0022 was examined. No statistically significant alterations in body weight were observed in animals treated with daily IP injections of 200 mg/kg of MS-0022 for 5 days (Figure 3G), indicating that the compound was well tolerated. Mice did not show any outward signs of toxicity or other side effects (weight loss, fur ruffling, hunched posture). Even though there were no outward signs of side effects or toxicity, we analyzed the AST/ALT levels as a measure of liver toxicity in xenografted animals that had been treated with MS-0022 for a longer time period than 5 days. As the liver normally acts as the primary site of drug metabolism, AST/ALT serum levels is an easily obtainable measure of toxic side effects. As seen in Figure 3C, AST/ALT levels remained greater than 1 in mice treated with 50 mg/kg of MS-0022 after a 30 days injection scheme (5 day injection, 2 day pause), suggesting that at this dose MS-0022 was well tolerated without apparent toxic effects in the liver in comparison to the control treated mice.

*In-vivo* efficacy of MS-0022 was tested against two pancreatic adenocarcinoma cell lines PANC-1 and SUIT-2. For PANC-1, subcutaneous tumors were established in CB17/SCID mice (n = 6). Mice were randomized and dosed with control solvent (water with 1% Tween 80) or MS-0022 (QD, IP at 50 mg/kg) in a 5 day injection/2 day pause scheme. Tumor volumes were evaluated throughout the treatment by measuring two perpendicular diameters with calipers, and by calculating tumor volume (mm^3^) using the formula V  =  a×b^2^× <Pi>/6. At the end of the treatment there was a 38% reduction in tumor volume and a 33% reduction in tumor weight compared to tumors in control solvent treated littermates (Figure 3H–I). The growth curve (Figure 3H), shows that the tumors in both control and treated groups eventually reached a similar growth pattern, and the growth inhibition by MS-0022 appeared to be transient. At the endpoint of the experiment, no statistically relevant difference in human (tumor) or mouse (stroma) Ptch1 or Gli1 mRNA levels could be identified in the extracted tumor mRNA (P>0.05) (Figure 3J).

**Figure 4:**
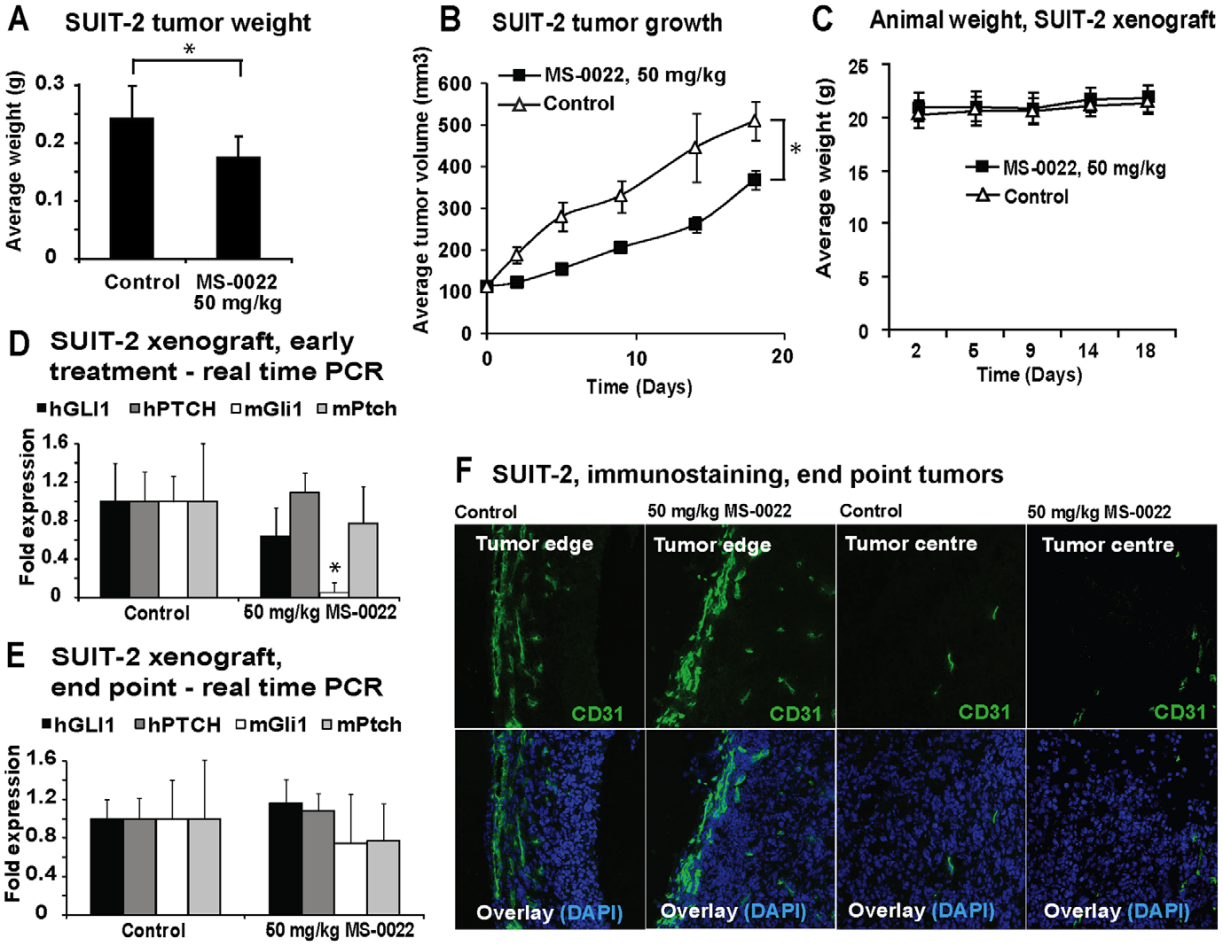
*In vivo* activity of MS-0022 in a SUIT-2 xenograft. A-F) Analysis of subcutaneous SUIT-2 tumors in mice treated with solvent control or 50 mg/kg MS-0022 for 7 (n = 3) or 18 days (n = 8) by PO administration. A) Graph displaying average SUIT-2 tumor weight at end of treatment, with SD. B) Tumor growth curve of SUIT-2 tumors in mice after 18 days of treatment, plotted as tumor volume (mm^3^) with standard error of the mean (SEM). C) Average animal weight through the course of the treatment with SD (n = 8). D) and E) Graphs showing normalized (GUSB) fold expression of mouse and human Gli1 and Ptch in treated tumors with SD. D) Shows expression in tumors harvested after 7 days (n = 3), while E) shows expression in tumors harvested after 18 days (n = 8). F) Immunostaining of microvessles using anti-CD31 (endothelial cell marker) in cryosectioned tumor tissue slides. Images depict CD31 staining in the tumor edge and in the centre of the tumors. *  =  P value <0.05.

Compared to PANC-1, SUIT-2 cells displayed a stronger responsiveness to MS-0022 *in vitro*, both in the reduction of GLI1 expression and in growth (MTS assay). Therefore, the effect of MS-0022 was also tested *in vivo* using SUIT-2 pancreas adenocarcinoma cells, and subcutaneous tumors were established in CB17/SCID mice (n = 11). Mice were randomized and dosed daily with solvent or 50 mg/kg MS-0022 via PO administration. The MS-0022 treatment did not result in any outwards signs of toxicity in the mice, and animal weight (Figure 4C) was unaffected by the treatment when compared to the animals that received control solvent. After 7 days, 3 mice from each treatment group were sacrificed and the tumors were harvested for analysis, while the remaining animals (n = 8) were sacrificed after 18 days of treatment, due to tumor burden. In general, the SUIT-2 tumors showed a more aggressive growth in comparison to the PANC-1 tumors. At the endpoint of the experiment, a 27% reduction in tumor volume and a 36% reduction in tumor weight were measured in treated animals compared to the control group (Figure 4A–B). Similar to PANC-1 xenografts (Figure 3D), a transient delay of growth was observed during the first days of treatment, followed by a recovery of growth with growth rates similar to untreated tumors. As in PANC-1 xenografts, no statistical relevant reduction of human or mouse Gli1, or Ptch1 mRNA levels was detectable at the endpoint of the experiment (Figure 4E). However, when samples from the SUIT-2 xenografts were analyzed after 7 days of treatment only, mouse (stromal) Gli1 mRNA in the tumors was reduced significantly (P<0.001), while both human and mouse PTCH and human GLI1 remained unaltered (P>0.05) (Figure 4D). There was no detectable difference in animal weight between the control and MS-0022 treated animals (Figure 4C). As inhibition of SMO has been linked to an increase in vascularization in poorly vascularized tumors [41], we investigated the vascularization of the tumor tissue upon MS-0022 treatment. Samples from the harvested tumors were cryosectioned, fixed and stained for the presence of the endothelial cell marker (CD31), as a marker for microvessles in tumor tissue. Large variations in SUIT-2 tumor shape including the occurrence of necrotic cavities, led to substantial variations in the vascularization of the tumors in all samples derived after 7 or 18 days of treatment and in control samples. Due to substantial variability, no apparent difference between the vascularization of samples derived from treated and untreated animals was detectable. Irrespective of MS-0022 treatment, CD31 staining revealed high vascularization at the tumor edge, while vascularization in the center of the tumor was generally low (Figure 4F).

## Discussion

MS-0022 was identified as a potent antagonist of Hh signaling that blocks the translocation of SMO to the cilia displaying a transient *in vivo* antagonistic effect in a pancreatic adenocarcinoma xenograft model.

MS-0022 contains a core structural motif common to other SMO antagonists. Analysis of the core structure of MS-0022 revealed that the “phenyl-amide-phenyl” (Figure 1A, where R3  =  phenyl) portion of the molecule was also present in HhAntag [33], GDC-0449 [52], Sant-2 [53] and Compound Z [54]. Deleting the 2-bromobenzene moiety of MS-0022 resulted in a total loss of activity, confirming the importance of the identified core structure for the activity of this class of molecules. Interestingly, although the “phenyl-amide-phenyl” core is identical in all of these molecules, there are also important differences in the structures. HhAntag, Sant-2 and Compound Z are closer to each other in structure than MS-0022 and GDC-0449, as they share a 1H-benzoimidazo structure coupled in the 3 position to the 4-chloro-N-phenyl. Interestingly, when the imidazo[1,2-a]pyridine of MS-0022 was deleted, activity was reduced. However, when it was substituted with groups that contain nitrogen in ortho position to the N-phenyl, partial activity was retained. Similar to GDC-0449 and cyclopamine, MS-0022 inhibited the Hh pathway activity after induction by various agonistic factors like Shh, oxysterols and SAG. The ability of MS-0022 to compete with BOPIDY-cyclopamine binding suggests that it acts by a similar mode of action as structurally related SMO antagonists, such as GDC-0449. This was further confirmed by the inhibition of SMO translocation to the primary cilium by MS-0022. While MS-0022 inhibited SMO in the nanomolar range, it displayed an additional effect on the Hh pathway downstream of SMO that required a higher dose. In the micromolar range, MS-0022 led to a significant reduction of Gli1 mRNA and protein levels in Sufu−/− cells. This effect was not due to an altered or inhibited translocation of Gli1 to the nucleus, indicating that MS-0022 did not reduce Gli1 levels by direct inhibition of Gli1. The precise cause of this secondary effect remains to be elucidated.

As the implications of aberrant Hh signaling are getting increasingly evident in several cancers, pathway antagonists will be an essential toolkit for development of future treatments. The emerging data from clinical trials with GDC-0449 show both the benefits and possible pitfalls of a pure SMO antagonist; a clear tumor response in Hh driven tumors such as basal cell carcinoma and medulloblastoma [34], [55], [56], and the occurrence of a drug induced resistance caused by mutations in the Smo locus [34], [55]. A possible solution to block Hh/GLI1signalling despite activation mutations in the Smo locus would be to develop drugs that act on the pathway downstream of SMO [47]. A GLI inhibitor would also address non-canonical activation of GLI [57].

The high level of tumor-stroma interactions found in advanced cancers like pancreatic adenocarcinoma poses additional challenges for developing treatment strategies based on Hh antagonists. Such strategies would require that, in addition to autocrine efficacy, paracrine interactions between tumor activated stroma and tumor cells are disrupted [33]. Furthermore, the barrier that the stromal cells create around the tumor, and the lack of compound delivery to areas of low vascularization need to be addressed [41].

The physiochemical properties of MS-0022 (MW  = 406.3; clogP: 5.44; PSA: 46.4 (Table 2)), its excellent per oral bioavailability, and the initial evidence that it is well tolerated *in vivo* (MTD >200mg/kg) makes MS-0022 a potential drug candidate. In PANC-1 and SUIT-2 xenograft experiments, MS-0022 treatment led to a partial response, where growth was halted during the first days of treatment compared to the control. Over time, however, both the treated and control xenograft groups reverted to similar growth. In both SUIT-2 and PANC-1 xenografts, the growth delay in the MS-0022 treated group resulted in a statistically relevant (P<0.05) average reduction in tumor volume of subsequently 27% and 38%. Our *in vivo* results lend support to the idea that the primary effect of Hh inhibition in pancreatic cancer treatment is due to the inhibition of Hh pathway activity in the stromal cells, although we also observe a mild initial reduction of GLI1 levels in tumor cells. Both pancreatic cancer cell lines, SUIT-2 and PANC-1, that were xenografted as a part of this study, express high levels of Shh mRNA (Table 4), and should be able to induce a Hh response in stromal cells. It^'^s interesting to note that though both MS-0022 and GDC-0449, and to a certain degree cyclopamine, led to a reduction of GLI1 mRNA in SUIT-2 cells *in vitro*, suggesting the possibility to affect autocrine Hh signaling in this cancer cell line. In an *in vivo* context, the effect of MS-0022 *in vivo* on SUIT-2 and PANC-1 tumors was most likely due to an inhibition paracrine Hh signaling since no statistically relevant reduction of either hGLI1 or hPTCH could be measured in mRNA derived from the tumor tissue. In contrast, mGli1 was reduced in the stroma of the SUIT-2 tumors (P<0.05) after 7 days of treatment, but not at the end of the treatment, while at the end of the treatment in PANC-1 xenografts, there was a slight reduction of mGli1 (although with a P>0.05). Reasons for the lack of a measurable reduction of hGLI1 or hPTCH in the tumors, at the time points that were taken, could be low levels of compound reaching the tumor, or activation mutations that occurred within the tumors. Activation mutations in SMO have been observed in a recent clinical trial using CDG-0449 in a medulloblastoma patient [34], [58]. Also, the development of resistance against chemotherapeutic agents in pancreatic cancer has been linked to both dense stromal matrix and increased stromal barrier, which over time, may cause resistance. Interestingly, Olive et al. found that treatment with IPI-926 led to an increased vascularization in KPC tumors, but similarly to our observations, the effect of this SMO inhibitor was due to a developing resistance [41].

KPC mice develop pancreatic tumors that resemble human pancreatic ductal adenocarcinoma due to a conditional expression of endogenous mutant Kras and p53 alleles in pancreatic cells [59], [60]. Similarly, the SUIT-2 and PANC-1 cell lines are mutant for p53 and Kras [61]–[65]. Despite the differences in the two models, with tumors forming in the mouse pancreas in the KPC model and tumor cells injected under the skin in the *in vivo* xenograft model, both studies provide evidence that the pancreatic cancer may only transiently be inhibited by a SMO antagonist, revealing that the challenge of overcoming resistance is ever present.

## Materials and Methods

### Ethics statement

All animals were housed and treated under the approved protocols in accordance with the National Institute of Health guide for the care and use of laboratory animals and according to regulations outlined in the USDA Animal Welfare Act (9 CFR Parts 1, 2 and 3) and the conditions specified in *The Guide for Care and Use of Laboratory Animals* (National Academy Press, Washington, D.C., 1996). Animal experiments performed in Norway were approved by the Centre for Comparative Medicine at the National Hospital of Norway according to the Regional Ethical Comity guidelines (permit nr: 19/08-910) and at the Norwegian Institute of Public Health (permit nr 2722), while animal experiments performed by Chemdiv were approved by ChemDiv's Institutional Animal Care and Use Committee (Permit nr: 8/21.01.2008). All efforts were made to minimize the number and suffering of animals.

### Cells and culture conditions

PANC-1, PC-3, THLE-2, NIH3T3, Shh-Light 2 (Shh-L2) and C3H10T1/2 cells were obtained from ATCC. SUIT-2 cells were obtained from Cell Bank, RIKEN BioResource Center. PTCH−/− MEF [45] was a gift from P. A. Beachy and SUFU−/− MEF were a gift from R. Toftgård (Karolinska Institutet). FEMX was a gift from G. E. Melandsmo. All cell lines were cultured at 37°C in a humidified atmosphere of 5% CO_2_ in the medium formulations as instructed by the suppliers.

### Alkaline phosphatase assay and screening protocol

C3H/10T1/2 cells were seeded 5*10^3^ cells per well in a 96-well format and allowed to attach for 3–5 hours, before adding serial dilutions of compounds (from a DMSO stock). After 30 minutes recombinant human SHH (C24II) (RnD systems (1845SH)) was added (300 ng/mL). After 72 hours, the cells were lysed for 15 minutes using 20 µL of a 10 mM Ethanolamine Buffer (pH 8.0) with 0.2% Triton-X100, supplemented 1∶100 with Protease Inhibitor Cocktail (EMD Bioscience). 7.5 µL of lysate was mixed with 45 µL of CSPD Alkaline Phosphatase Substrate with Emerald-II (Applied Biosystems) and incubated at room temperature, in the dark, for 45 minutes. The plate was measured using a Wallac VictorV reader (0.1 s Luminescence). Screening compounds and MS-0022 were part of a Chemdiv chemical library.

### Hh reporter assay

Shh-L2 cells were used as a Hh reporter cell line as described previously [45]. Hh signaling was induced by Shh conditioned medium, 200 nM SAG (AH-Diagnostics) and ActOXS (a combination of 5 µM Hydroxycholesterol 20-alfa and 5 µM Hydroxycholesterol 22S (Sigma-Aldrich)). For Shh conditioned medium, Shh-PANC-1 cells were grown to confluence and switched to fresh medium. Shh conditioned medium was harvested after 48 hours, and was diluted to 50% in fresh DMEM before use. The clonal Shh-PANC-1 cell line was generated by stably transducing PANC-1 cells with virus containing medium from HEK293T cells that were transfected using a Virapower Lentiviral expression kit (Invitrogen). The vector was produced by removing GFP from a pLenti 6.2-vector, and inserting the mouse Shh cDNA sequence.

### IC50 calculation

IC50 values were calculated using dose response curve data where n = 3. The calculations were performed with an online calculator using the formula: **a exp (−bx) + c** at the website; http://www.changbioscience.com/stat/ec50.html

### NMR spectroscopy of MS-0022

NMR spectra of MS-0022 (approx. 2 mg) were obtained from a solution in 0.5 ml of DMSO-*d*6 ((CD_3_)_2_S(O), 99.9% D, Cambridge Isotope Laboratories, Andover, MA) in a 5.0 mm tube (WILMAD, WG-5 Economy). The spectra were acquired on an Avance AV 600 MHz NMR spectrometer (Bruker BioSpin, Rheinstetten, Germany) with a 5 mm CP-TCI (1H/13C, 15N-2H) triple-resonance inverse cryo probe, equipped with a Z-gradient coil. NMR assignments of MS-0022 were inferred from examination of ^1^H- and ^13^C spectra, attached proton test (APT), correlated spectroscopy (COSY45), total correlation spectroscopy (TOCSY) pulsed field gradient heteronuclear single quantum coherence (g-HSQC), pulsed field gradient heteronuclear multiple bond correlation (g-HMBC) and pulsed field gradient heteronuclear two-bond correlation (g-H2BC/g-HSQC-COSY). The data were processed using the Bruker TOPSPIN software (version 1.3 or version 2.1 pl 2). Chemical shift values were referenced to the residual solvent signals, i.e. C*H*D_2_S(O)CD_3_ = 2.49 ppm and (*C*D_3_)_2_S(O)  = 39.5 ppm, respectively. All NMR spectra were acquired at 25°C.

### BODIPY assay

BODIPY assay was performed to determine competition with cyclopamine as described previously [21], [66].

### Real Time PCR

Cells were lysed for RNA extraction after 48 or 72 hours of treatment. Total RNA from cultured cells or tumor tissue was isolated using the GeneElute miniprep kit (Sigma-Aldritch) following the manufacturer^'^s instructions. cDNA was synthesized from the isolated mRNA using the Retroscript kit (Ambion), and real-time PCR was carried out using the SYBR Green PCR master mix (Stratagene) according to the manufacturer^'^s instructions with an Mx3000P cycler (Stratagene). The relative concentrations of cDNA present in each sample were calculated by the MxPro software (Stratagene), normalized for GAPDH (mRNA from cells) or GUS (mRNA from tumors). For real time PCR primer sequences see Table 6. For the tumor tissue real time PCR, previously described human and mouse specific primers against GUSB, PTCH1 and GLI1were used [33].

**Table 6:** Primer sequences for quantitative real time PCR.

Primers:	Primer sequence, 5′ → 3′	Tm,°C
hPTCH1 fwd	CGA GCC CCC CTG TAC GAA GTG G	67.7
hPTCH1 rev	GAC CCC CAG CAA GCC CAG AAA A	64.0
hGLI1 fwd	GCG CAT CCC GAG CCC AGC	65.1
hGLI1 rev	GCC CTC GGT GCA GCT GTT GGT C	67.7
hGLI2 fwd	GCC TCC GAG AAG CAA GAA GCC AAA A	64.4
hGLI2 rev	CCT GGT GTC GCA TGT CAA TCG GTA G	66.3
hGLI3 fwd	CGG GAC GGT GTT TGC CAT GGA C	65.8
hGLI3 rev	GGA GGA TGG AAG GCA GGG AAA AGA T	64.6
hSHH fwd	GCC AGC GGA AGG TAT GAA GGG AAG	66.1
hSHH rev	ACC GAG ATG GCC AAA GCG TTC AAC	64.4
hHIP fwd	TGG GGA TGG CTC GCA ACG TCT C	65.8
hHIP rev	TGG GAT GGA ATG CGA GGC TTA GC	64.2
hSUFU fwd	CCC GAG GAT GAC GAG GAC AGC C	67.7
hSUFU rev	CGC GTG CGA ATC AGC TCA TGG G	65.8
hSMO fwd	CCA GGA GGA AGC GCA CGG CAA G	67.7
hSMO rev	TCG CAC TGG CCT GAA CTG TTG AAC T	64.6
hGAPDH fwd	GCC CCC TCT GCT GAT GCC CCC A	75.7
hGAPDH rev	TGG GTG GCA GTG ATG GCA TGG	70.2
mGLI1 fwd	AGC CAA CTT TAT GTC AGG GTC CCA GGG T	71.2
mGLI1 rev	GAG CCC GCT TCT TTG TTA ATT TGA CTG	66.5
mGAPDH fwd	TAT GTC GTG GAG TCT ACT GGT GTC TTC ACC	68.1
mGAPDH rev	GAG TTG TCA TAT TTC TCG TGG TTC ACA CCC	66.8

Table of real time PCR primers; name, sequence and Tm°C (h =  human, m = mouse).

### Immunofluorescence

PTCH−/− MEF were seeded on pre-coated glass slides (1 hour coating with 0.1% Gelatin (G1393) and 0.003% Collagen (C8919) (Sigma-Aldrich) in PBS). 80% confluent cells were switched from 10% to 0.5% FBS in medium and cells were treated with DMSO or 5 µM MS-0022 for 24 hours in (n = 3) before subsequent immunofluorescent staining: Slides were fixed with 4% paraformaldehyde (Sigma-Aldrich), permeabilized for 10 min in 0.1% v/v Triton X-100 (Sigma-Aldrich) in PBS (PBT), and blocked with 10% w/v BSA (Saveen Werner) in PBT for 1 hour. Primary antibodies, mouse anti-acetylated tubulin (a-Tub) (Sigma-Aldrich (T7451), 1∶1000) goat anti-Smo (Santa Cruz Biotechnology (sc-6367), 1∶1000), and goat anti-GLI-1 (RnD systems (AF3455), 1∶500) were diluted in 0.5% w/v BSA in PBT and left on the slides over night at 4°C. Secondary antibodies donkey anti-goat IgG-Alexa 594 and donkey anti-mouse IgG-Alexa 488 (Invitrogen, both at 1∶500) were added in 0.5% w/v BSA in PBT for 1 hour. The slides were counterstained with DAPI (Roche, 1 mg/ml) in PBS. 3 random regions were imaged pr slide using a confocal LSM510 microscope (Carl Zeiss MicroImaging). The ratio of green Smo positive cilia versus the red aTub positive cilia was quantified manually or processed and analyzed using ImageJ to quantify the intensity of Smo and aTub for each cilium in the images (http://rsb.info.nih.goc/ij/). Graphs display mean with standard deviation error bars.

Frozen tumor tissue was cryosectioned (n = 8 pr group), and the sections were fixed with 4% paraformaldehyde for 10 minutes before permeabilization with 0.2% Triton-X in PBS for 10 minutes. Sections were blocked for 30 minutes at room temperature with 3% BSA in PBS and was subsequently incubated over night at 4°C with rat anti-mouse CD31 (BD Biosciences (550274), 1∶500) in 1% BSA in PBS. The secondary antibody used was Alexa Fluor 488 goat-anti-rat (Molecular Probes; 1/700 dilution) in 1% BSA in PBS for 1 hour at RT. Nuclei were counterstained with DAPI, in PBS for 10 minutes at RT. Images was acquired as above and electronic images was further processed using ImageJ.

### Transfection and luciferase assay

Vectors were obtained from the following: GliBS-Luc reporter [67] (gift from H. Sasaki), pBluescript (pBS) (Stratagene), FL-HA-GLI1 (Gli1) and Gli2-GFP (Gli2) (gifts from R. Toftgård), Super8XTOPFlash [68], and NFkB-luc (Panomics). 10 ng renilla luciferase (pRL-TK) (Promega) was used as a transfection control. HEK293 and NIH3T3 cells were seeded at 80,000 per well in 48-well plates on day 1, and were co-transfected on day 2 using a total of 0.4 µg plasmid per well mixed at 1∶3 in FUGENE6 according to manufacturer^'^s instructions. On day 3, control medium and activating medium (50% L1 conditioned medium, 50% Shh conditioned medium or medium containing 10 ng/mL recombinant rcrTNF-α (R&D Systems)) +/− 10–20 µM MS-0022 were added, using DMSO as a control (the NFkB pathway inhibitor Bay11-7082 was used as a control in the NFkB assay). Samples were analyzed on day 4 or 5 using the Dual Luciferase assay kit according to manufacturer^'^s instructions. Graphs display relative or fold reporter activity, and are calculated from a mean ratio of firefly reporter luminescence/pRL-TK luminescence with standard deviation error bars (n = 3). The experiments have been repeated with similar results.

### *In vitro* antiproliferative assay

Cells were plated at a density of 3000 (THLE-2 and PC-3) and 1000 (PANC-1, SUIT-2 and FEMX) cells per well (on the basis of their growth rate) in 200 µl of medium in a 96-well plate. Medium was changed daily, and after four days, the assay was read using a MTS kit (Promega) according to the instructions of the manufacturer. Graphs display average of percent growth with standard deviation (n = 8).

### Passage growth assay

20,000 cells were seeded per well in 6 well plates (triplicates) with DMSO or 5 µM MS-0022. Treatment medium was changed on day 3–5, and after 5–7 days the cells were trypsinized, re-suspended in medium and counted, before reseeding cells at 20,000/well for a subsequent passage. The average growth of MS-0022 treated cells was calculated as a percentage of the growth of the DMSO control at the end of each passage. Graphs display average of percent growth with standard deviation bars (n = 3).

### Soft agar colony formation assay

2000 PANC-1 or SUIT-2 cells were suspended in 1.5 mL growth media containing 0.35% agar (Oxoid), over a 1.5 mL base layer containing 0.5% agar in 6-well plates. The plates were incubated for 14 days (SUIT-2) and 21 days (PANC-1) and then stained for 1 h with 1 ml of 0.02% crystal violet (Sigma-Aldrich), and colonies were counted according to sizes; <0.2 mm (small), 0.2–1 mm (medium) and >1 mm (large). Data represent average numbers with standard deviation bars (n = 3). The experiments have been repeated with similar result.

### Pharmacokinetics, dose tolerance, animal tumor establishment and treatment

For pharmacokinetic (PK) analysis, 5 mg/kg MS-0022 was given by IV, PO and IP administration to 7–8 week old male C57BL mice (*mouse musculus*), and blood was collected after 5, 15, 30, 60, 120, and 240 min, with n = 3. Animals were sacrificed and blood samples (∼0.5 mL each) were collected from the abdominal aorta into EDTA-containing tubes, centrifuged (2–8°C for ∼10 minutes at ∼10000 xg) and plasma harvested into single tubes for each animal and frozen (∼−70°C). Blood samples were analyzed by ChemDiv^'^s bioanalytical department. The maximum concentration (Cmax), total drug exposure (the area under the curve to the last quantifiable concentration (AUClast) and as the AUClast value extrapolated to infinity (AUCinf), calculated as AUCinf = AUClast+C(t)last/Kel, where C(t)last is the last measurable concentration), half-life (T1/2) calculated as ln(2)/Kel and Kel calculated as the slope of the terminal linear portion of the concentration/time curve. The WinNonlin Professional 5.2 software (Pharsight Corp.) was used for the calculation of the PK parameters.

For the analysis of uptake of MS-0022 in organs and tissue, livers were dissected out from mice 120 min after PO administration of MS-0022 and frozen (∼−70°C). The LC-MS method used to detect MS-0022 was described previously [69], with the main exception being that isocratic conditions were used (50% 0.1% FA (*aq*), 50% 0.1% FA (ACN), v/v), and that a UV detector was placed between the LC and MS instrument.

MS-0022 tolerance was tested by daily injections of 200 mg/kg MS-0022 for 5 days in 7–8 week old male C57BL mice. Animal were observed daily for outward signs of toxicity (weight loss, fur ruffling, hunched posture).

For PANC-1 xenografts, 4–6 week old CB17/SCID mice were injected with 5×10^6^ PANC-1 (n = 6 per group) cells in 200 µl PBS subcutaneously on day 1. IP treatment with 50 mg/kg MS-0022 began when median tumor size reached 25 mm^2^ (day 27). MS-0022 was pulverized using a pestle, and was mixed into a stable suspension in water containing 1% Tween 80 (Sigma-Aldrich). Mice were treated with MS-0022 or solvent for a period of 30 days after initiation in a 5 day injection 2 day pause scheme, and tumor size was measured throughout using calipers (mm^2^). After 30 days the mice were sacrificed and tumors dissected and weighed. The PANC-1 xenograft endpoint was taken two days after the last compound treatment.

For SUIT-2 xenografts, 4–6 week old CB17/SCID mice were injected with 5×10^6^ SUIT-2 (n = 11 per group) cells in 200 µl PBS subcutaneously on day 1. IP treatment with 50 mg/kg MS-0022 began when median tumor size reached 35 mm^2^ (day 8). Mice were treated daily with MS-0022 or solvent for a period of 18 days after initiation, and tumor size was measured throughout using calipers (mm^2^). After 8 days, three mice from each group were sacrificed and tumors dissected, weighed and processed for analysis. The remaining animals (n = 8) were sacrificed after 19 days of treatment, and tumors harvested. Tumor volume (mm^3^) was calculated using the formula a×b^2^× <pi>/6. End tumor measurement statistics were obtained using two samples T-test in the Sigmaplot software.

### Liver transaminases

Blood was collected from the hepatic vein of freshly sacrificed animals into heparinized capillary blood collection tubes (Sarstedt), which were immediately centrifuged and the serum was collected. AST and ALT were run on non-hemolyzed samples using AST and ALT kits (Randox) following the manufacturer^'^s instructions.

## Supporting Information

Figure S1 **Atom numbering of MS-0022.** The atom numbering of MS-0022 used in the ^1^H and ^13^C NMR analysis. (See Table S1)
(TIF)

Table S1 **NMR analysis of MS-0022.** ^1^H and ^13^C NMR Data for MS-0022 (DMSO-d_6_). For the atom numbering used see Figure S1.
(DOC)
