# Supplementary figures and images for: A Novel Synthetic Smoothened Antagonist Transiently Inhibits Pancreatic Adenocarcinoma Xenografts in a Mouse Model
Source: PLoS One. 2011 Jun 15;6(6):e19904. doi: 10.1371/journal.pone.0019904 (PMC3115942; doi:10.1371/journal.pone.0019904)

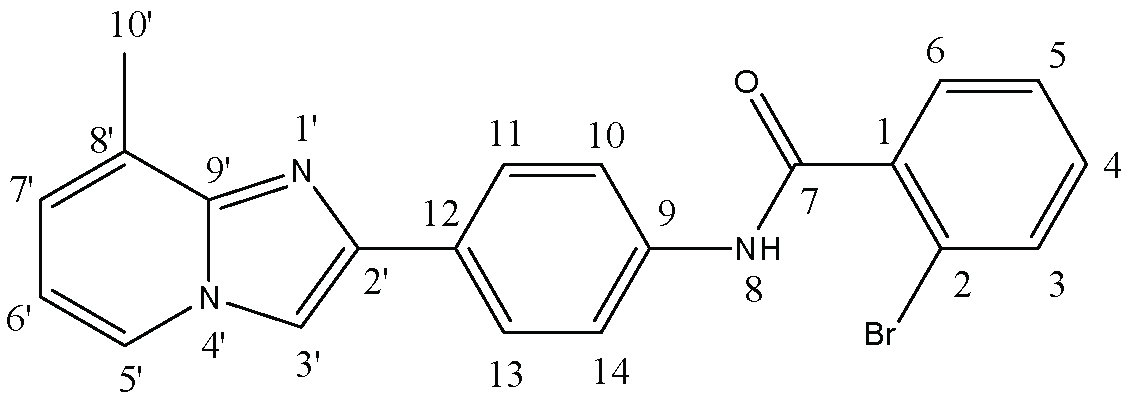

Supplement: Figure S1 — Atom numbering of MS-0022. The atom numbering of MS-0022 used in the 1H and 13C NMR analysis. (See Table S1) (TIF) [file pone.0019904.s001.tif]
