# Supplementary material for: A Novel Synthetic Smoothened Antagonist Transiently Inhibits Pancreatic Adenocarcinoma Xenografts in a Mouse Model
Source: PLoS One. 2011 Jun 15;6(6):e19904. doi: 10.1371/journal.pone.0019904 (PMC3115942; doi:10.1371/journal.pone.0019904)
Supplement: Table S1 — NMR analysis of MS-0022. 1H and 13C NMR Data for MS-0022 (DMSO-d6). For the atom numbering used see Figure S1. (DOC) [file pone.0019904.s002.doc]

| Position | δH (*J* in Hz) (Recorded at 600 MHz) | δC |
| --- | --- | --- |
| 1 |  | 139.1 |
| 2 |  | 119.0 |
| 3 | 7.72, d (8.1) | 132.8 |
| 4 | 7.42, dd (8.1, 7.7) | 131.2 |
| 5 | 7.50, dd (7.7, 7.4) | 127.8 |
| 6 | 7.57, d (7.4) | 128.9 |
| 7 |  | 165.8 |
| 8 | 10.56, br s |  |
| 9 |  | 138.4 |
| 10 | 7.79, d (8.6) | 119.8 |
| 11 | 7.95, d (8.6) | 126.0 |
| 12 |  | 129.7 |
| 13 | 7.95, d (8.6) | 126.0 |
| 14 | 7.79, d (8.6) | 119.6 |
| 1' |  |  |
| 2' |  | 143.6 |
| 3' | 8.32, s | 109.2 |
| 4' |  |  |
| 5' | 8.35, d (6.7) | 124.6 |
| 6' | 6.78, dd (6.9, 6.7) | 112.2 |
| 7' | 7.03, d (6.9) | 123.4 |
| 8' |  | 126.0 |
| 9' |  | 145.3 |
| 10' | 2.52, s | 16.7 |
